# Luminal Conversion and Immunoregulation by Probiotics

**DOI:** 10.3389/fphar.2015.00269

**Published:** 2015-11-12

**Authors:** Bhanu Priya Ganesh, James Versalovic

**Affiliations:** ^1^Department of Pathology and Immunology, Baylor College of Medicine, Houston, TX, USA; ^2^Department of Pathology, Texas Children’s Hospital, Houston, TX, USA

**Keywords:** probiotics, metabolites, commensal bacteria, immunomodulation, diet, dietary compounds, microbiome

## Abstract

Beneficial microbes are responsible for the synthesis of nutrients and metabolites that are likely important for the maintenance of mammalian health. Many nutrients and metabolites derived from the gut microbiota by luminal conversion have been implicated in the development, homeostasis and function of innate and adaptive immunity. These factors clearly suggest that intestinal microbiota may influence host immunity via microbial metabolite-dependent mechanisms. We describe how intestinal microbes including probiotics generate microbial metabolites that modulate mucosal and systemic immunity.

## Introduction

The mammalian gastrointestinal tract, site for digestion and nutrition absorption harbors commensal microbiota, a population composed of 1000–5000 different bacterial species. Metagenomics of the Human Intestinal Tract (MetaHit) project containing 249 newly sequenced samples with 1,018 previously sequenced samples were combined to create a cohort from three continents. From this the integrated gene catalog (IGC) comprising 9,879,896 genes were established. The catalog includes close-to-complete sets of genes for most gut microbes. Analyses of a group of samples from Chinese and Danish individuals using IGC revealed country-specific gut microbial signatures. This expanded catalog should facilitate quantitative characterization of metagenomic, metatranscriptomic, and metaproteomic data from the gut microbiome to understand its variation across populations in human health and disease (Qin et al., 2010; Ferreira et al., 2014; Li et al., 2014). Recent studies show that changes in the commensal bacterial composition are linked to various metabolic and inflammatory diseases including inflammatory bowel disease (IBD; Sokol et al., 2008), obesity and type 2 diabetes (Everard et al., 2013; Dao et al., 2015), allergy (Berni Canani et al., 2015), and colorectal cancer (Swidsinski et al., 1998). These interrelationships provoke multiple fundamental questions regarding the cellular and molecular pathways through which commensal microbiota regulates mammalian gene expression and influence a wide range of clinically important diseased complications. The intestinal microbiota affects host physiology in many ways such as influencing the maturation of the immune response and fortifying the intestinal barrier against pathogenic bacteria. Importantly, intestinal microbes are potential regulators of digestion converting a wide range of non-digestible carbohydrates to short chain fatty acids (SCFA), which can be absorbed by the host and used as energy sources (Sharma et al., 2010; Becker et al., 2011).

Dysregulation of intestinal immune response by commensal microbiota plays an important role in the onset and development of different immune-mediated disorders (Wohlgemuth et al., 2009; Feng et al., 2010). For example, the presence of *Akkermansia muciniphila*, commensal mucin degrader, has been shown to exacerbate *Salmonella* Typhimurium infection by worsening intestinal inflammation, increasing macrophage infiltration and elevating proinflammatory cytokines in gnotobiotic mice (Ganesh et al., 2013). Flagellin-detecting toll like receptor 5 (TLR5) knockout mice colonized with adherent-invasive *Escherichia coli* (AIEC) during microbiota acquisition drove chronic colitis. AIEC instigates chronic inflammation by increasing microbiota levels of LPS and flagellin (Chassaing et al., 2014). Recent findings described how commensals are recognized by the intestinal innate immune system and how individual species can influence specific modules of the innate and adaptive immunity. Germ-free mice were shown to have fewer and smaller Peyer patches, exhibit a local defect or absence of TH1, TH17, and TREG cells, and their intestinal epithelia express lower amounts of TLRs and MHC class II, as compared with mice that have been exposed to normal microbiota (commensals). Similarly, symbiosis factor polysaccharide A (produced by *Bacteroides fragilis*) can induce TREG cells and suppress TH17 cells via engagement of TLR2 on CD4^+^ T cells (Round et al., 2011). Similarly, another human commensal *Faecalibacterium prausnitzii* suppresses IL-8 production and NF-κB signaling in response to inflammatory secretion of IL-1β (Sokol et al., 2008). Altogether, recent evidence has provided insights into immune-mediated mechanisms in metabolic disorders (Borchers et al., 2009). Taken all the findings together, existing data argues for the need to probe the microbiome for new strategies for immunomodulation, either by enhancing (immunodeficiency) or by suppressing (allergy) host immunity. Microbial metabolites and nutrients derived from beneficial bacteria in the intestine via luminal conversion may modulate host immunity and profoundly affect mammalian biology of the “holobiont.”

## Changes in Microbial Diversity and Treatment with Probiotics

**FIGURE 1:**
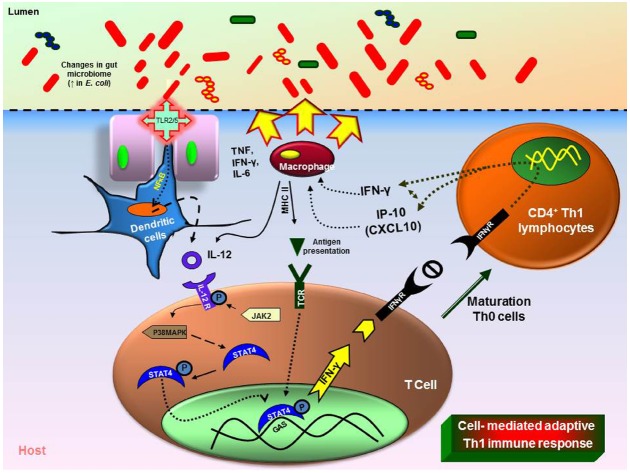
**Immune responses triggered by changes in the gut microbiome.** Intestinal inflammation in the UC or CD leads to dysbiosis (imbalance microbiota). Overgrowth of enteropathogenic bacteria causing increased activation of toll-like receptors (TLR) 2 or 4. This causes the activation and translocation of nuclear factor kappa B (NFκB) and causes secretion of pro-inflammatory cytokine interleukin (IL)-12. Increased IL-12 causes T-helper (Th) Th1/Th2 immune response with increase in tumor necrosis factor (TNF), IL-6, interferon gamma (IFN-γ). The dysbiosis leads to increase in immune cells (macrophages, neutrophils) at the infected site causing severe inflammation (MHCII—major histocompatibility complex).

Recent studies in rodents show that inflammation and/or infection is correlated with changes in bacterial composition (Packey and Sartor, 2009; Saulnier et al., 2011; Pflughoeft and Versalovic, 2012; Ganesh et al., 2013). Molecular techniques are clarifying changes in the composition of the mucosal associated and fecal microbiota in patients with IBD esp., ulcerative colitis (UC), and Crohn’s diseases (CD) together with widely expanding previous culture based studies. Patients with UC and CD have decreased complexity of commensal microbiota revealed by examining DNA libraries (Frank et al., 2007). More specifically, members of the phyla Bacteroidetes and Firmicutes are decreased in CD and UC patients (Backhed et al., 2005). A member of the family Firmicutes, *F. prausnitzii* was reduced in the patients with CD and this was confirmed and associated with increased risk of post-resection recurrence of ileal CD (Frank et al., 2007; Sokol et al., 2008; Swidsinski et al., 2008). *In vitro* peripheral blood mononuclear cell stimulation by *F. prausnitzii* decreased pro-inflammatory cytokines IL-12 and IFN-γ and stimulated secretion of anti-inflammatory cytokine IL-10. Oral administration of live *F. prausnitzii* or its supernatant reduced the inflammation severity by TNBS and corrected the associated dysbiosis (Baumgart et al., 2007). However, the abundance of *E. coli* is increased in IBD patients (Figure 1; Kotlowski et al., 2007). Similarly, the mucosal *E. coli* numbers *in situ* correlates with the severity of ileal disease and invasive *E. coli* are restricted to inflamed mucosa. Finally, fecal and mucosal associated microbial communities of UC and CD patients are consistently less diverse with increased instability. Commensal non-pathogenic bacteria can cause colitis in host with immunomodulatory and mucosal barrier deficits. Interleukin (IL)-10^–/–^ germ-free mice colonized with *Enterococcus faecalis* and/or invasive *E. coli*, showed aggressive TH1/TH17-mediated colitis within 3 weeks but this was not observed in the WT mice. LPS from microbes were detected by dendritic cells (DCs). DCs play an important role through antigen presentation via TLRs in linking between the innate and adaptive immunity (McKenna et al., 2005). DCs are the initial cells to synthesize IL-12 under well characterized microbial stimulants of the cytokines. IL-12 selectively promotes the differentiation of Th1 CD4^+^ cells upon stimulation with antigens (de Jong et al., 2002). Th1 cell-mediated immune response leads to the paradigm of T-helper cell differentiation in which IL-12 cytokine mediated activation of STAT4 and is critical for generation of Th1 cells (Kaplan et al., 1998). IL-12 mediated immune response is dependent upon the presence of CD4^+^ and CD8^+^ T lymphocytes and upon the production of IFN-γ finally causing cell-mediated adaptive immunity (Figure 1; Kim et al., 2007). However, certain class of bacteria like probiotic bacterium, *Bifidobacterium breve* increased IL-10 secretion Tr-1 cells in the colon and inhibits inflammation (Jeon et al., 2012). Introducing such beneficial strains in an unhealthy intestinal environment will potentially be a novel therapeutic strategy.

Most importantly, metabolites produced by intestinal microbiota have direct effects on the host mucosa. Commensal bacterial fermentation of non-digestible fiber leads to increased luminal bioavailability of SCFAs like butyrate, acetate, fumarate, and propionate (Cummings and Macfarlane, 1997). Bacterial metabolites such as butyrate serve as potential energy sources for colonic epithelial cells, whereas other fermentation by-products like hydrogen sulfide (HS), nitric oxide (NO) and proteases produced by subsets of commensals may enhance histopathology. Butyrate metabolism by colonic epithelial cells might be suppressed by HS/NO metabolites, resulting in starvation of colonocytes and yielding histopathology similar to that of UC (Roediger et al., 1993; Packey and Sartor, 2009; Cain and Karpa, 2011). The butyrate producing probiotic bacterium *Clostridium butyricum* MIYAIRI 588, increase the butyrate availability in the presence of fibrous diet (Weng et al., 2015). Intracellular butyrate and propionate (but not acetate) has been shown to inhibit the activity of histone deacetylases (HDACs) in colonocytes and immune cells, which promotes the hyperacetylation of histones, in addition to some transcription factors and proteins that are involved in signal transduction. This has multiple consequences for gene expression and cellular differentiation, including the down-regulation of pro-inflammatory cytokines, such as IL-6 and IL-12, in colonic macrophages and is also known to inhibit colorectal cancer (Louis et al., 2014). Similarly, pretreatment of *Helicobacter pylori*-induced gastric ulcers with *C. butyricum* in mice showed significantly reduced numbers of mucosal lesions with decreased quantities of proinflammatory cytokines (Wang et al., 2015). Probiotics may provide beneficial functions into the GI tract which might enhance the functionality of the existing commensal communities. Probiotics may also affect the composition of the intestinal microbiota by providing colonization resistance and competition for nutrients or production of pathogenic inhibitors and modulates intestinal immune response.

Probiotics possess the ability to transiently colonize the gut (Valeur et al., 2004; Ukibe et al., 2015; Vieira et al., 2015) and facilitating proliferation of commensal microbes, while enhancing microbial diversity (Sherman et al., 2009). Probiotics are known to exert antimicrobial effects as a front line of defense against the luminal pathogens. For example, some probiotics are known to elaborate some microbial products known as bacteriocins. These probiotic factors can inhibit the growth and virulence of enteric bacterial pathogens (Corr et al., 2007). *Bifidobacterium animalis* subsp. *lactis* (*B. lactis*), *Streptococcus thermophilus*, two different strains of *Lactobacillus delbrueckii* subsp and *L. lactis* subsp in fermented milk were used to determine the impact of microbes in a mouse model of IBD. The findings show that *B. lactis* containing fermented milk decreased cecal pH, altered SCFA concentrations, increased the relative quantities of lactate- and butyrate-consuming bacteria, and reduced intestinal inflammation scores (Veiga et al., 2010). In addition, lactic-acid producing bacteria are known to exert antimicrobial effects on pathogens by reducing the pH of the microenvironment in the lumen of the GI tract (Fayol-Messaoudi et al., 2005). Probiotics or their metabolites reduced the secretion of immunomodulation molecule autoinducer-2 by the pathogenic *E. coli*, which results in reduced gene expression contained in the locus of enterocyte effacement (Pathogenicity Island) which is critical for mediating intimate bacterial binding to the host cell surfaces, called attachment and effacing lesion (Mack et al., 1999; Russell et al., 2007). *Lactobacillus plantarum* has been shown to have the capacity to enhance the production and secretion of mucins esp. MUC2 and MUC3 from the human intestinal epithelial cells (Mack et al., 1999), which improves the epithelial barrier function (Corfield et al., 1992, 2000). Similarly, bacteria and their by-products may have direct effect on the betterment of host health.

## Luminal Conversion of Dietary Components by the Intestinal Microbiota

**FIGURE 2:**
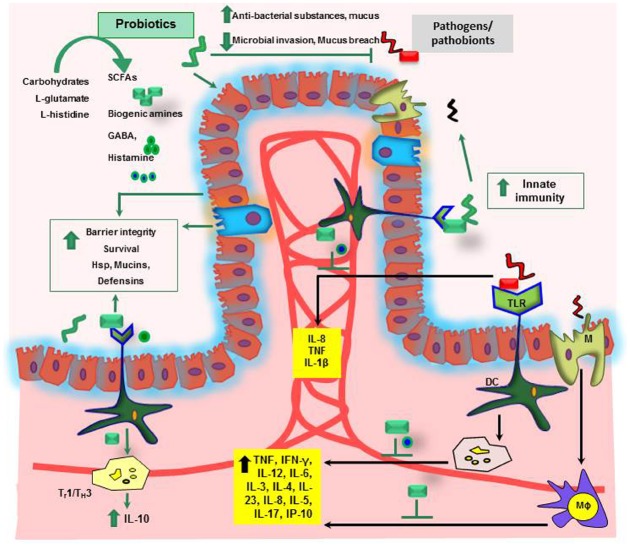
**Mechanisms of probiosis in the gastrointestinal tract.** SCFAs, short chain fatty acids; GABA, gamma-aminobutyric acid; Hsp, heat shock proteins; IL, interleukin; TNF, tumor necrosis factor; Th, T-helper; IFN-γ, interferon gamma; Mϕ, Macrophage; DC, dendritic cell; M, microfold cells.

Human diet may have a direct impact on the intestinal microbiota which ultimately leads to the changes in the microbiota composition. These changes have been recently validated using mouse model experiments. Mice subjected to the high fat diet in obese mice showed major changes in microbial composition with an increased proportion of the phylum Firmicutes and decreased proportion of Bacteroidetes. In particular, species like *Clostridium ramosum* was correlated with increased body weight (Fleissner et al., 2010; Woting et al., 2014). Vitamins, amino acids or dietary fibers with the diet are assimilated and converted into other metabolites in the lumen by intestinal microbiota. Some of the products of these bio-chemical conversions were SCFA, biogenic amines (such as histamine) or other amino acid derived metabolites like serotonin or gamma-aminobutyric acid (GABA; Bravo et al., 2011; Figure 2) which may have beneficial effect on host health (Hemarajata and Versalovic, 2013; Hemarajata et al., 2013). Serotonin is a neurotransmitter, biochemically derived from tryptophan (Best et al., 2010). *Bifidobacterium infantis* colonization in rats modulated the bioavailability of tryptophan by yielding increased concentrations of tryptophan in plasma, reduced 5-HIAA (hydroxyindoleacetic acid) concentrations in the frontal cortex, and diminished quantities of 3,4-dihydroxyphenylacetic acid (DOPAC) in the amygdaloid cortex (Desbonnet et al., 2008). Gut microbial populations in SPF mice modulated brain development by contributing to suppressed expression of postsynaptic density protein (PSD)-95 and synaptophysin in the striatum compared to germ-free mice (Diaz Heijtz et al., 2011). Treatment with *Bifidobacterium* species resulted in normalization of the immune response, reversal of behavioral deficits, and restoration of basal noradrenaline concentrations in the brainstem, thereby alleviating depression of the CNS (Desbonnet et al., 2010). In addition, orally gavaged BALB/c mice with *Lactobacillus rhamnosus* (JB-1) reduced GABA_*A*α2_ gene expression in the prefrontal cortex and amygdala, but increased GABA_*A*α2_ gene expression in the hippocampus. These findings provide evidence that *Lactobacillus* strains regulate emotions, behavior and central GABA receptor expression (Bravo et al., 2011). Intestinal microbiota may modulate the bioavailability of tryptophan in the intestine, and may in turn influence availability of neurotransmitters such as serotonin in the host. Non-digestible carbohydrates can be fermented in the lumen resulting in production of SCFAs such as lactate, formate, acetate, propionate, butyrate and valerate (Blaut, 2013). These metabolically active SCFAs are involved in various biological processes as an energy source in intestinal epithelial cell proliferation (Astbury and Corfe, 2012; Fung et al., 2012; Matthews et al., 2012). Additionally, fermentation of prebiotic carbohydrates such as inulin and fructo-oligosaccharides has been shown to increase the proportion of beneficial microbes like *Bifidobacterium* spp. and *Lactobacillus* spp. in the obese mice and was negatively correlated with serum entoxin levels (Salazar et al., 2014). Consumption of western diet showed increased level of plasma LPS concentration and this was correlated with increased changes in microbiota composition (Cani et al., 2013; Everard and Cani, 2013; Everard et al., 2013). Moreover, a recent study shows that dietary plant lignans were converted to estradiol like metabolite enterodiol and enterolactone by intestinal bacteria in germ-free rats colonized with lignan-converting consortium, such as *Clostridium saccharogumia*, *Blautia producta*, *Eggerthella lenta*, and *Lactonifactor longoviformis*. The produced enterolignans suppressed tumor number and tumor cell proliferation in hormone related cancer (Mabrok et al., 2012).

The secondary plant metabolites, glucosinolates from Brassica vegetables, were converted to isothiocyanates (glucosinolate derivative) and were measured in urine, luminal contents and plasma of mice (Budnowski et al., 2013). In addition, glucosinolates and their derivatives have been shown to reduce AOM/DSS induced colon carcinogenesis in mice (Lippmann et al., 2014). For example, *Bacteroides thetaiotaomicron* isolated from human fecal sample can convert glucosinolates into isothiocyanates, measured in luminal contents of rats (Elfoul et al., 2001; Krul et al., 2002), and these compounds potentially suppress lung cancer cell metastasis by inhibiting cell survival signaling molecules Akt and NFκB activation in human lung large cell carcinoma (Wu et al., 2010b). Similarly, isoflavones have been implicated in the prevention of hormone-dependent and age related diseases, including cancer (Birt et al., 2001; Scalbert et al., 2005; Geller and Studee, 2006; Usui, 2006). Intestinal bacteria, e.g., *Slackia isoflavoniconvertens*, play an important role in the metabolism of isoflavones, daidzein and genistein to equol (Chang and Nair, 1995; Rafii et al., 2003; Matthies et al., 2008, 2012). Based on the structural similarities of these bacterial by-products with estrogens, they bind to estrogen receptors and thus may prevent cancer progression (Matthies et al., 2008; Lepri et al., 2014).

## Immunomodulation by Probiotics

Probiotics (beneficial microbes) are frequently, though not necessarily be a commensal bacteria. Probiotics are defined as “beneficial live micro-organisms which when administrated in adequate amounts confer beneficial effects on the host health” (Mack et al., 1999; Peran et al., 2006; Borchers et al., 2009; Ganesh et al., 2012; Isolauri et al., 2012; Klaenhammer et al., 2012; Thomas et al., 2012; Morelli and Capurso, 2012; Arena et al., 2014; Dylag et al., 2014; Galdeano et al., 2015; Ki et al., 2014; Repa et al., 2014; Sah et al., 2014; Sanders et al., 2014). Most known probiotics until now are either lactobacilli or bifidobacteria representatives of which are normal inhabitants of the gastro-intestinal (GI) tract (Blum et al., 2002; Wohlgemuth et al., 2009). Recently, animal experiments and human studies suggest that therapeutic manipulation of the balance between beneficial and detrimental intestinal bacterial species can influence health and disease (Fitzpatrick, 2013). The known mechanisms of probiosis include manipulation of intestinal microbial communities, suppression of pathogens, immunomodulation, activation of anti-apoptotic genes in human or mouse intestinal epithelial cells from cytokine induced apoptosis, differentiation and fortification of the intestinal barrier (Thomas and Versalovic, 2010). For example, simultaneous treatment with probiotic *Streptococcus thermophilus* ATCC19258 and *Lactobacillus acidophilus* ATCC 4356, prevent invasion of entero-invasive *E. coli* and enhance the intestinal epithelial barrier function by amplifying the phosphorylation of occludin and ZO-1 together with a reduction of pro-inflammatory responses *in vitro* (Resta-Lenert and Barrett, 2003). Another similar study also demonstrated that application of probiotic *E. coli* NISSLE (EcN) is able to mediate up-regulation of ZO-1 expression in murine IECs and confer protection from the Dextran sodium sulphate (DSS) colitis-associated increase in mucosal permeability to mice luminal substances (Ukena et al., 2007).

Loss of tolerance to the patient’s own commensal microbiota has been implicated in the development of IBD (Wu et al., 2010a). Use of probiotics, to shift the existing microbiota balance in favor of protective microbial species and to treat IBD, has been extensively reviewed (Ochoa-Reparaz et al., 2009). The ability of some probiotics to synthesize bacteriocins (Awaisheh et al., 2013) or to induce the secretion of antibacterial cryptidins by Paneth cells (Hooper et al., 2003; Ayabe et al., 2004) could account for such changes in microbiota composition or even for the protection against pathogenic bacteria. In addition to the effects mediated by bacteria–bacteria interactions, probiotics may have a direct effect on host physiology. In the inflamed gut, the down-regulation of pro-inflammatory cytokines by probiotics may be an important factor for the observed improvement of symptoms (Figure 2; Ma et al., 2004). For example, *Lactobacillus casei* DN-114001 treatment increases the number of CD4^+^FoxP3^+^ regulatory T cells in mesenteric lymph nodes (mLN), decreases the production of the pro-inflammatory cytokines TNF-α and IFN-γ, changes the gut microbiota composition and prevents DSS induced colitis in BALB/c mice (Zakostelska et al., 2011). However, only few molecular mechanisms underlying probiotic action have so far been identified. Activation of TLR9 by bacterial DNA has been proposed as one possible mechanism of a probiotic-mediated amelioration of experimental colitis (Rachmilewitz et al., 2004). TLRs belong to highly conserved receptors of the innate immune system. TLR activation results in the translocation of the nuclear factor NFκB into the cell nucleus triggering transcription of immunorelevant genes (Cario and Podolsky, 2005). In addition, *L. casei* inhibits post-transcription of pro-inflammatory interferon γ-induced protein 10 (IP-10) in intestinal epithelial cells of colitic IL-10 knock-out mice (Hormannsperger et al., 2009).

An intact intestinal epithelial cell layer is of utmost importance for preventing the uncontrolled intrusion of pathogenic bacteria. However, pathogenic bacteria are capable of compromising the integrity of the epithelium by disrupting the tight junctions between epithelial cells (Berkes et al., 2003). Bacterial factors improving epithelial integrity have been identified for the probiotic *Lactobacillus* GG. This strain produces two soluble proteins (p40 and p75) which protect epithelial cells from apoptosis and thereby increase mucosal integrity. The secreted proteins activate anti-apoptotic protein kinase B (PKB/Akt) in a phosphatidylinositol-3′-kinase (PI3K)-dependent pathway and inhibit the pro-apoptotic p38/mitogen-activated protein kinase (MAPK; Yan et al., 2007).

**FIGURE 3:**
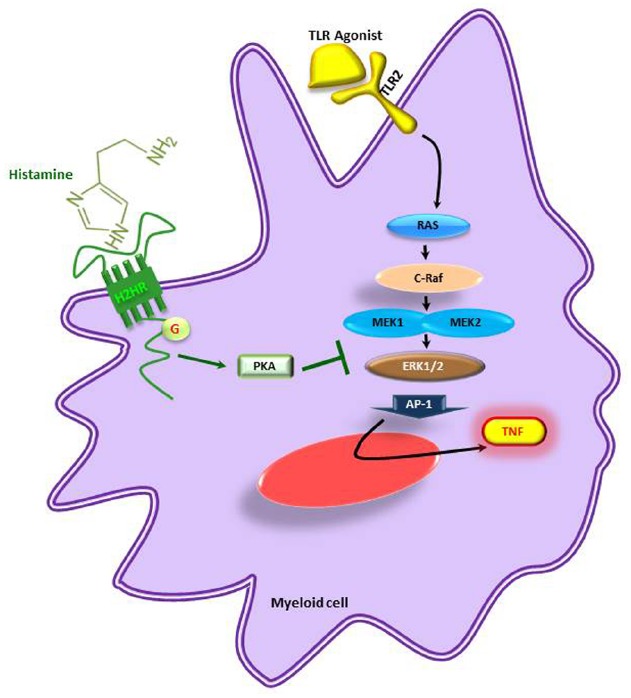
**Microbe-derived histamine mediated suppression of pro-inflammatory cytokines.** TNF is suppressed by inhibition of the MEK/ERK pathway in myeloid cells. H2HR, histamine receptor 2; PKA, activated protein kinase A; TNF, tumor necrosis factor; TLR, toll-like receptor. Adapted from Thomas et al. (2012).

Similarly, the biogenic amine, histamine, produced by decarboxylation of amino acid L-histidine by histidine decaxboxylase gene cluster (*hdc*) in *Lactobacillus reuteri* ATCC 6475 showed immunomodulatory effects by suppressing TNF production in myeloid progenitor cell lines (Figure 3) whereas the *L. reuteri* lacking *hdc* gene cluster was unable to suppress the pro-inflammatory cytokine TNF. The bacterial derived histamine binds to and activates histamine receptor H2 (HRH2) and there by inhibits MEK/ERK MAPK signaling pathway and presumably suppress TNF transcription and Ap-1 translocation (Thomas et al., 2012). These findings clearly demonstrate that bacterial interactions directly or indirectly have an impact on host physiology. Therefore, in the current review we mainly focused on the different beneficial bacteria and their metabolites on immunoregulation of the host.

## Conclusion

In the presented review we demonstrated how probiotic bacteria or their metabolites regulate immunomodulatory effects on the host health. Probiotics have been proposed as preventive and therapeutic measures in order to restore the healthy microbiota composition and function of the GI tract. Additionally restoring the current balance is very important because the commensal bacteria are important source of vitamins, amino acids and lipid homeostasis and alternation in the levels of these metabolites might have an influence on the immune system (Brestoff and Artis, 2013). Therefore, therapeutic manipulations of intestinal bacteria by selectively altering the beneficial versus detrimental species by probiotics and or prebiotics administration could reverse the inflammatory responses and restore mucosal homeostasis. Future challenges include interrogations of molecular mechanisms through nutrients and beneficial bacterial metabolites, regulate immune response and linking the commensal bacteria-beneficial probiotic bacteria-metabolite-immune system axis in the content of health and diseases, may provide useful insights for the development of improved, preventive and therapeutically cost-effective and non-toxic approaches to treating different disorders mainly IBD.

### Conflict of Interest Statement

The authors declare that the research was conducted in the absence of any commercial or financial relationships that could be construed as a potential conflict of interest.
